# The Moving Junction Protein RON8 Facilitates Firm Attachment and Host Cell Invasion in *Toxoplasma gondii*

**DOI:** 10.1371/journal.ppat.1002007

**Published:** 2011-03-10

**Authors:** Kurtis W. Straub, Eric D. Peng, Bettina E. Hajagos, Jessica S. Tyler, Peter J. Bradley

**Affiliations:** 1 Department of Microbiology, Immunology and Molecular Genetics, University of California, Los Angeles, Los Angeles, California, United States of America; 2 Department of Microbiology and Immunology, Stanford University School of Medicine, Stanford, California, United States of America; University of Georgia, United States of America

## Abstract

The apicomplexan moving junction (MJ) is a highly conserved structure formed during host cell entry that anchors the invading parasite to the host cell and serves as a molecular sieve of host membrane proteins that protects the parasitophorous vacuole from host lysosomal destruction. While recent work in *Toxoplasma* and *Plasmodium* has reinforced the composition of the MJ as an important association of rhoptry neck proteins (RONs) with micronemal AMA1, little is known of the precise role of RONs in the junction or how they are targeted to the neck subcompartment. We report the first functional analysis of a MJ/RON protein by disrupting *RON8* in *T. gondii*. Parasites lacking RON8 are severely impaired in both attachment and invasion, indicating that RON8 enables the parasite to establish a firm clasp on the host cell and commit to invasion. The remaining junction components frequently drag in trails behind invading knockout parasites and illustrate a malformed complex without RON8. Complementation of *Δron8* parasites restores invasion and reveals a processing event at the RON8 C-terminus. Replacement of an N-terminal region of RON8 with a mCherry reporter separates regions within RON8 that are necessary for rhoptry targeting and complex formation from those required for function during invasion. Finally, the invasion defects in *Δron8* parasites seen *in vitro* translate to radically impaired virulence in infected mice, promoting a model in which RON8 has a crucial and unprecedented task in committing *Toxoplasma* to host cell entry.

## Author Summary

Apicomplexan parasites actively invade host cells to survive, with an important step being the formation of a tight interface between parasite and host cell membranes called the *moving junction* (MJ). Passing over the length of the invading parasite, the MJ anchors the pathogen to enable propulsion into a parasitophorous vacuole (PV) formed from host membrane. This structure also selectively filters transmembrane proteins from the membrane surrounding the PV, preventing its targeting to host lysosomes. The MJ's molecular nature is understood as an association between proteins secreted from rhoptry and microneme organelles, but the functional significance of the rhoptry neck (RON) components that predominate within this complex is entirely unknown. Our study describes the first functional analysis of any MJ/RON protein in *Toxoplasma*, RON8. RON8 knockout parasites are severely deficient in both attachment and entry, likely due to the inability of the parasite to firmly engage the host cell. When *Δron8* parasites do invade, MJ proteins are often secreted in disorganized trails, indicating the MJ is unstably formed without RON8. From this data, we propose that loss of RON8 produces a crippled parasite frequently incapable of firm attachment, drastically retarding the establishment of vacuoles *in vitro* and subsequent disease *in vivo*.

## Introduction

Host cell invasion by apicomplexan parasites is a highly specialized feature of this phylum that is crucial to their survival in humans (*Toxoplasma gondii, Plasmodium spp.*, *Cryptosporidium spp.*) and animals (*Neospora caninum*, *Eimeria spp*.) [1], [2]. Following initial contact with a target host cell via GPI-anchored parasite surface proteins, the pathogen reorients its apical end toward the host cell membrane and secretes the contents of microneme, rhoptry, and dense granule secretory organelles in an intricate, coordinated manner to enable parasite invasion and concomitant formation of a unique vacuole within the host [3].

A key stage in invasion is the formation of a tight apposition between parasite and host cell membranes known as the MJ, first visualized in electron micrographs of invading *Plasmodium* [4]. Initially a punctate focus, this interface rapidly resolves into a ring that slides posteriorly over the parasite in conjunction with host membrane invagination and eventual engulfment of the invading pathogen. The MJ is essential for this process, as it anchors the parasite to the host surface while the parasite's actin-myosin motor (the “glideosome” [5]) provides forward motion into the host cell. In addition, studies of invading *Toxoplasma* parasites using fluorescently labeled lipids and host membrane proteins have demonstrated molecular sieving at the junction, presumably responsible for the non-fusogenic nature of the PV that precludes destruction by host lysosomes [6], [7]. Although the mechanism by which the MJ carries out these functions is unknown, the identification of a complex of rhoptry neck proteins that specifically localize to the *Toxoplasma* MJ provided a significant advance in the characterization of the unique invasion process used by apicomplexan parasites [8], [9], [10], [11].

Rhoptries are subdivided into mottled bulbous bodies and tapered electron-dense necks, corresponding to storehouses of proteins that play distinct roles in invasion [12]. Rhoptry bulb proteins (ROPs) are injected into the host cell where they contribute to the formation of the parasitophorous vacuole [13] and co-opt host cell processes to create a favorable environment for the parasite [14], [15]. Several neck proteins, by contrast, assemble with the microneme protein AMA1 to constitute the *Toxoplasma* MJ [8], [10]. Orthologs of most known *Toxoplasma* MJ/RON components localize to the *Plasmodium* rhoptry neck [16], [17], [18], [19] and traffic to the *Plasmodium* moving junction [20]. While this establishes the generally conserved nature of the complex across the Apicomplexa, MJ proteins lack identifiable domains or motifs that could provide clues to their function in this enigmatic invasion machinery.

 A novel rhoptry neck protein, RON8, was recently identified in *Toxoplasma* and *Neospora* as a coccidia-specific component of the MJ [9], [11]. RON8 associates with RONs 2/4/5 in a preformed complex within the rhoptry necks that is injected into the MJ with RONs 4/5/8 exposed to the cytoplasmic face of the host cell membrane tethered to RON2, which is thought to span the host plasma membrane via its transmembrane domains [9]. This topology could be facilitated by RON2′s integration within multi-lamellar whorls detected in *Plasmodium* rhoptries by electron microscopy, which could insert into the host plasma membrane during invasion, enabling soluble RONs bound to RON2 to be exposed to the host cytoplasm [21]. Here they are ideally poised to carry out filtration or anchoring roles for the parasite, as molecular sieving by the moving junction is restricted to this face of the host membrane [6], [7]. These findings are supported by exogenous expression of RON8 within mammalian cells, as RON8 in the absence of other complex members traffics to its site of action at the cell periphery via its C-terminal region [11]. In addition to cleavage of the signal peptide, RON8 is processed at the N-terminus, like other MJ/RONs, and the proforms of these proteins can associate *in vitro* with each other and with pro-AMA1 [9]. AMA1 coprecipitates RON2 under harsh conditions and likely has a favored association with this RON, supporting a model where the universal “receptor” RON2 grants *Toxoplasma* access to a wide variety of host cells through binding its universal “ligand,” AMA1, lodged in the parasite membrane [9]. Aside from establishing contact between RON2 and AMA1, however, precise roles for the MJ/RON proteins during invasion remain elusive.

In this study, we present RON8 as the first RON junction component to be disrupted in *Toxoplasma*, and show that RON8-deficient parasites have severe defects in host cell entry. Junction proteins secreted from invading *Δron8* parasites are often disorganized, suggesting an imperfect separation of the PV from the host cell membrane after invasion. We further show through complementation of knockout parasites that the RON8 C-terminus is processed, and also identify functional domains of RON8 that are sufficient for rhoptry neck targeting and MJ complex association. RON8-ablated parasites are greater than five logs less virulent in mice, reinforcing the devastating consequences of lacking RON8 for infection *in vivo* and subsequent formation of disease. Taken together, this work identifies crucial adherence and organizational roles for RON8, demonstrating the importance of this protein within the junction for committing *Toxoplasma* to host cell entry.

## Results

### Deletion of RON8 from *Toxoplasma*

Our initial attempts to disrupt *RON8* from the RH strain of *T. gondii* were not successful [11], consistent with the necessity of the MJ complex for parasite invasion [8], [22]. The recent development of *Toxoplasma* strains lacking the non-homologous end-joining protein KU80 virtually eliminates heterologous DNA insertion and enables highly efficient gene knockouts [23], [24]. We therefore investigated whether this strain would be more receptive to a direct deletion of RON8.

**Figure 1:**
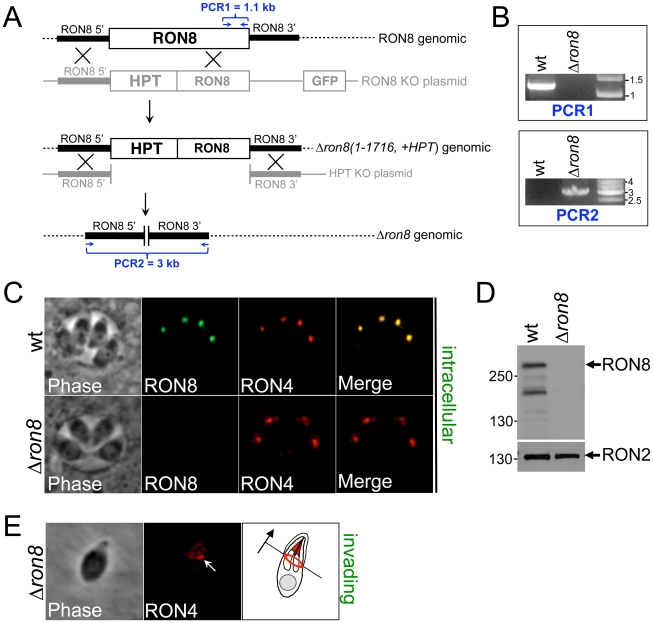
RON8 is the only RON/MJ protein to be disrupted in the Apicomplexa. **A**) Schematic of the two-step targeting strategy used to ablate *RON8* from *Δku80*Δ*hpt* parasites. Homologous recombination at the *RON8* locus replaced the portion of the gene encoding residues 1-1716 with selectable marker *HPT* and concurrently removed the negative screening marker *GFP*. This generated the intermediate strain *Δron8 (1-1716, +HPT)*. To remove the remaining *RON8* genomic sequence and the *HPT* cassette, a 2^nd^ targeting construct without *HPT* and a 3′ flank downstream of the *RON8* stop codon was homologously inserted into the *Δron8 (1-1716, +HPT)* genome, producing *Δron8* parasites. PCR1 amplifies sequence inclusive of C-terminal exons, while PCR2 amplifies sequence between the regions upstream and downstream of the *RON8* locus. (**B**) PCR1 confirms the loss of *RON8* coding sequence from the *Δron8* strain, and PCR2 establishes the bridging of RON8 flanking regions through removal of *RON8*. **C**) IFAs of wildtype (*Δku80Δhpt* parental) and *Δron8* intracellular parasites show the absence of RON8 staining in the rhoptry necks of knockout parasites. RON4 is used for colocalization to the rhoptry necks for each strain. **D**) Western analysis of lysates made from wildtype and *Δron8* parasites demonstrates the loss of RON8 expression in the knockout strain, where RON2 serves as a loading control. An ∼230 kDa major RON8 breakdown product previously seen [11] is also visible. E) The moving junction can still be detected in invading *Δron8* parasites, as seen by RON4 staining (white arrow). The cartoon illustrates the direction of invasion for this *Δron8* parasite (black arrow).

The RON8 deletion construct was transfected into *Δku80Δhpt* parasites [25], (referred to as wildtype strain) to replace the sequence encoding residues 1-1716 with the selectable marker hypoxanthine-xanthine-guanine phosphoribosyl transferase (HPT) (Figure 1A). We observed that parasites lacking RON8 in transfected populations were fully outcompeted by drug-resistant *RON8+* parasites in less than four passages, consistent with a defect in invasion. We were able to isolate a clonal line of knockout parasites by cloning early following transfection, named *Δron8 (1-1716, +HPT*) strain parasites. To remove the remainder of the *RON8* locus and the *HPT* selectable marker, a second construct (*HPT KO*, Figure 1A) was transfected into *Δron8 (1-1716, +HPT)* parasites. Following negative selection with 6-thioxanthine and cloning, PCR confirmed both the absence of RON8 coding sequences and the concomitant shortening of the distance between *RON8* 5' and 3′UTRs normally separated by ∼20 kb (Figure 1B). We thus generated *Δku80ΔhptΔron8* parasites, referred to as *Δron8* (Figure 1A).

Immunofluorescence assays (IFA) of intracellular *Δron8* parasites demonstrated that RON8 was not present in the rhoptry necks (Figure 1C), and Western blot analysis confirmed this strain to be devoid of RON8 (Figure 1D, note that in lysates of wildtype parasites an ∼230 kDa breakdown product of RON8 is frequently seen as previously described [11]). RON4 could still be detected in the moving junction of invading knockout parasites (Figure 1E, arrow), demonstrating that the ability of these parasites to form junctions was not completely compromised by the loss of RON8. We observed that higher inoculums were necessary for obtaining infection levels similar to wildtype parasites; however, no gross change in the time needed for intracellular parasites to replicate was observed (data not shown). While these results establish RON8 is not absolutely required for propagation of *Toxoplasma*, the rapid loss of *Δron8* parasites from transfected populations suggested a severe invasion defect in parasites lacking this junction component.

### Complementation of *Δron8* parasites by targeted insertion at the *KU80* locus

**Figure 2:**
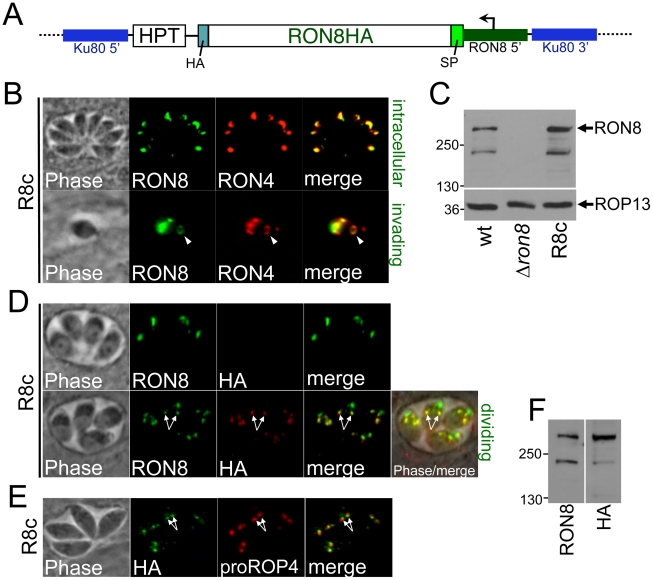
Complementation of *RON8* by targeting a RON8 expression cassette to the *KU80* locus. **A**) Diagram of the complementation construct used to generate R8c parasites. The HA-epitope-tagged RON8 coding sequence is driven by ∼1.8 kb of RON8 promoter sequence (dark green) and GRA2 3'UTR (not shown) with the downstream selectable marker *HPT.* The construct was targeted to the ablated *KU80* locus by homologous recombination using KU80 flanking regions (blue). **B**) R8c parasites demonstrate restored RON8 expression in the rhoptry necks of intracellular parasites (as shown by colocalization with RON4 by IFA, top panel) and restored RON8 traffic to the moving junction during invasion (arrowhead, bottom panel). **C**) Western analysis of wildtype, *Δron8*, and R8c parasite lysates indicates slightly greater levels of RON8 expression in complemented parasites than the wildtype strain; equal lysate loads are shown by the ROP13 control (bottom panel). **D**) Immunofluorescence showing that the C-terminal HA tag of R8c parasites is often not detected in intracellular parasites (top panel). In parasites where HA is detected, RON8 colocalization shows two more posterior spots that are suggestive of pro-rhoptries (arrows, bottom panel). **E**) Detection of the C-terminal HA tag in the pro-rhoptries of R8c parasites is confirmed by IFA colocalization (arrows) by colocalization with anti pro-ROP4 antibodies. **F**) Comparison of the size of RON8 (detecting primarily the mature form) and HA (detecting the unprocessed form in the pro-rhoptries) via Western blot reveals no detectable shift in size between the unprocessed form and the mature form of the protein. This indicates that the C-terminal cleavage event does not remove a large portion of this region of the protein.

Complementing knockout parasites at the *RON8* locus would not strictly eliminate the possibility that the observed defect in invasion was due to polar effects resulting from the ablation of the gene. Given that *Δku80* parasites greatly favor homologous integration of transfected DNA, we executed a novel strategy for complementing *Δron8* by targeting a RON8 expression cassette containing a C-terminally tagged version of RON8 driven from its endogenous promoter to the ablated *KU80* locus (Figure 2A). A clonal line of complemented parasites (referred to as R8c) showed exogenous RON8 both trafficked correctly to the rhoptry necks of intracellular parasites (Figure 2B) and localized to the moving junction during invasion (Figure 2B, arrow). Western blot analysis demonstrated slightly higher levels of RON8 expression in this line compared to wildtype parasites (Figure 2C). PCR confirmed integration of the RON8 expression cassette at the *KU80* and not the *RON8* locus (not shown). Thus, the *KU80* locus appears to be a suitable site for complementation in this recently-developed strain of *T. gondii* parasites.

### RON8 undergoes C-terminal processing

We observed that the C-terminal HA tag on RON8 was frequently not detectable in intracellular R8c parasites by immunofluorescence (Figure 2D, *top panels*). When HA was observed, it did not colocalize with RON8 in the parasite apex but instead stained two slightly more posterior dots indicative of pro-rhoptries formed in dividing parasites (Figure 2D, *bottom panels,* arrows). The HA staining within these posterior dots colocalizes with the pro-form of ROP4 in R8c parasites (Figure 2E, arrows) [26], confirming pro-rhoptry localization for HA-tagged RON8 and indicating C-terminal processing of the protein during rhoptry development. To investigate the extent of proteolytic cleavage, we compared the size of the protein detected by anti-RON8 antibodies with that given by anti-HA (Figure 2F). No distinguishable difference in size was observed between these two forms of the protein, suggesting that the cleavage site does not lie far from the extreme C-terminus, although determination of the extent of cleavage is difficult given RON8's large size (∼330 kDa).

### RON8 is an important mediator of firm attachment and commitment to invasion of host cells

**Figure 3:**
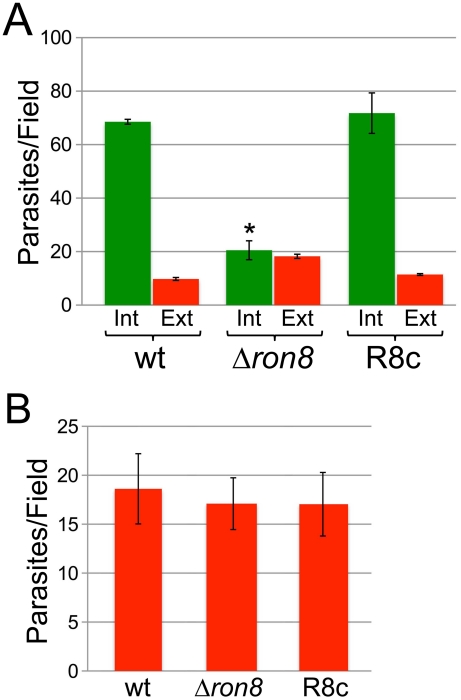
Parasites lacking RON8 are deficient in invasion likely through increased detachment from host cells. **A**) Quantification of invasion using red/green assays demonstrates a substantial invasion defect for *Δron8* parasites that is rescued upon complementation. Green bars represent internal/penetrated parasites, while red bars depict attached/extracellular parasites for wildtype, *Δron8*, or R8c strains allowed to invade fibroblast monolayers for 1 hour. For each strain, at least 250 total parasites were counted from nine random fields per sample, and values are presented as internal (Int) or external (Ext) parasites per field. Data are mean values +/− SEM (error bars) for two independent experiments performed in triplicate. The asterisk indicates that parasite penetration is significantly lower (p value = 0.0245 using a Student's two-tailed t-test) in Δ*ron8* parasites compared to wildtype. **B**) Initial stages of attachment are unaffected in Δ*ron8* parasites. Equal numbers of wildtype, *Δron8*, or R8c parasites were preincubated with 1 µM cytochalasin D for 15 min prior to incubation with host fibroblasts in the presence of cytochalasin D, then fixed and stained in detergent-free conditions with rabbit antisera against SAG1. For each strain, values are displayed as total numbers of parasites counted divided over nine random fields. The data is expressed as mean values +/− SEM for two independent experiments performed in triplicate.

To examine defects in invasion displayed by *Δron8* parasites in detail, we performed red/green invasion assays [27], in which wildtype, *Δron8*, or R8c parasites were allowed to infect for a one-hour time period and extracellular and intracellular parasites were detected by staining and microscopic counting. A dramatic 70% reduction in penetration was observed in knockout parasites compared to the wildtype control, a defect which is completely reversed in R8c parasites (Figure 3A). While we anticipated seeing a concomitant increase in attached parasites that failed to invade in the *Δron8* strain, we intriguingly saw only a mild increase in the number of extracellular parasites (Figure 3A). This could be due to a failure of the knockout parasites to attach to host cells, a function previously associated solely with microneme proteins. To assess whether gross perturbation of microneme function was detectable in *Δron8* parasites, we examined the localization of MIC2 and also assessed the levels of both parasite-associated and secreted MIC2 and saw no apparent differences (Figure S1A–C). We additionally assessed gliding motility by examining deposits of parasite surface antigens onto FBS-coated slides from wildtype versus knockout parasites and again saw no noticeable differences (Figure S1D). While we cannot exclude the possibility that loss of RON8 impacts other microneme functions, these data suggest that microneme protein generation and function of the actin-myosin motor central to driving *T. gondii* invasion are not grossly affected in *Δron8* parasites.

Although RON8 contributions to the initial stages of host cell attachment would explain lower than expected counts of extracellular *Δron8* parasites, this protein could alternatively be important for maintaining a secure contact with host cells, the absence of which leads to abortive invasion and subsequent parasite detachment. To differentiate between these possibilities, we incubated wildtype, *Δron8*, or R8c parasites in cytochalasin D (cytD) to permit only the initial stages of attachment, thereby isolating these steps from the rest of invasion (Figure 3B) [29]. Similar levels of attachment were observed for all three strains, suggesting that RON8-deficient parasites can properly attach, but do not form a *stable* grip and eventually release the host cell. As treatment with cytD does not inhibit the secretion of rhoptry bulb proteins in evacuoles [29], we also stained for these vesicles using anti-ROP2/3/4 antisera. We counted noticeably fewer evacuoles secreted from knockout parasites than either wildtype or R8c strains suggesting that the knockout parasites are less able to advance to the stage at which they are committed to invasion, although variability in numbers between experiments precluded assessing statistical significance (not shown). Together, these experiments demonstrate that invasion is impacted both at the steps of parasite attachment and entry, and that the attachment defect is likely due to parasites failing to obtain the firm grip on host components that is necessary for a commitment to host cell entry.

We have previously shown that exogenously expressed RON8 traffics to the periphery of host cells, its predicted site of action during invasion. We tested whether host-expressed RON8 could complement the knockout by comparing infections of wildtype and Δ*ron8* parasites in cells expressing RON8, but saw no apparent rescue of the invasion defect (data not shown). This is most likely due to the inability to incorporate RON8 into the rest of the complex during the rapid process of parasite entry, but also could be due to differences in processing or other modifications of the parasite-derived form of RON8 that are not present in the exogenously expressed protein.

### Loss of RON8 produces abnormal secretion trails of junction proteins

**Figure 4:**
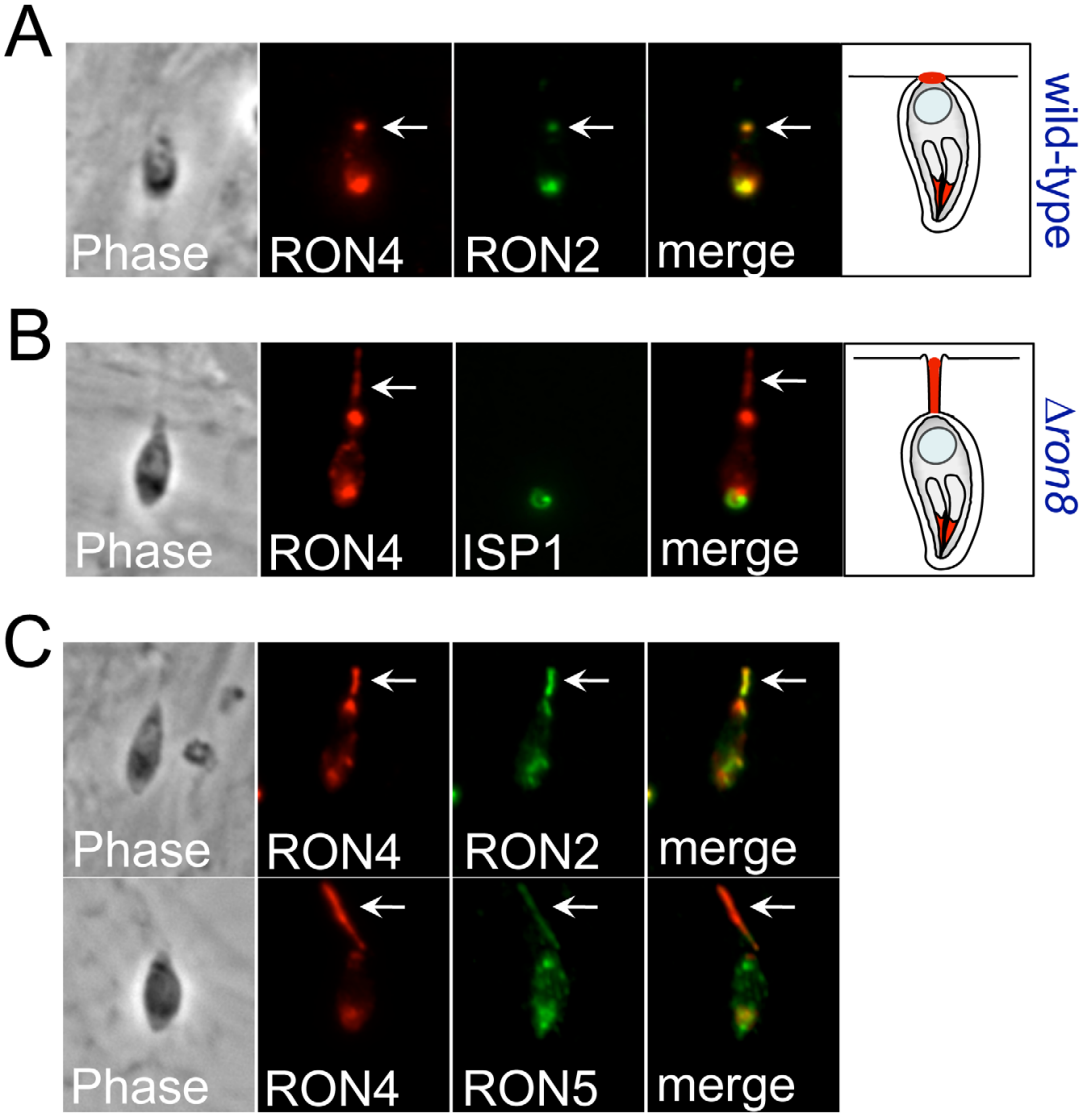
Disorganized secretion of junction components in Δ*ron8* parasites. **A**) Pulse invasion assays using wildtype parasites were carried out for 5 min on host fibroblasts prior to IFA with antibodies against RON4 and RON2, showing the typical punctate spot of junction proteins (arrow) after the parasite has fully invaded the host cell but the vacuole has likely not yet detached from the host membrane (see cartoon). **B**) Similar assays conducted with *Δron8* parasites show a trail of RON4 secreted from the posterior end of the newly invaded parasite (arrow). The orientation of the parasite is shown by costaining with anti-ISP1 which detects the apical cap of the IMC. **C**) Other MJ/RONs detected by anti-RON2 (top panel) or anti-RON5C antibodies which (bottom panel) colocalize with RON4 in secreted trails deposited by *Δron8* parasites (arrows).

Having established the diminished ability of *Δron8* parasites to invade host cells, we used immunofluorescence to further examine the junction morphology in those knockout parasites that could successfully invade. Whereas the junction appears as a punctate residual focus on the PV membrane of newly invaded wildtype and R8c parasites (Figure 4A), ∼15% (15% and 16% in two independent experiments) of *Δron8* parasites displayed short trails of RON4 extending from the posterior end upon entering the host cell (arrows in Figure 4B). In addition to RON4, the other MJ/RONs RON2 and RON5C also specifically localized to these trails (Figure 4C). The trails of MJ components are distinct from so called “slime trails” deposited by gliding parasites on FBS coated slides as the MJ components are not present in slime trails or in staining of extracellular parasites. The trails also appear to be specific to RON/MJ proteins as the non-junction rhoptry proteins (ROP2/3/4) are not present within these structures (data not shown). The appearance of these trails in the absence of RON8 suggests this protein is important for maintaining the integrity of the MJ complex or for pinching the nascent PV off from the plasma membrane at the end of invasion.

### Selective complementation reveals regions of RON8 sufficient for rhoptry neck targeting and MJ complex formation

**Figure 5:**
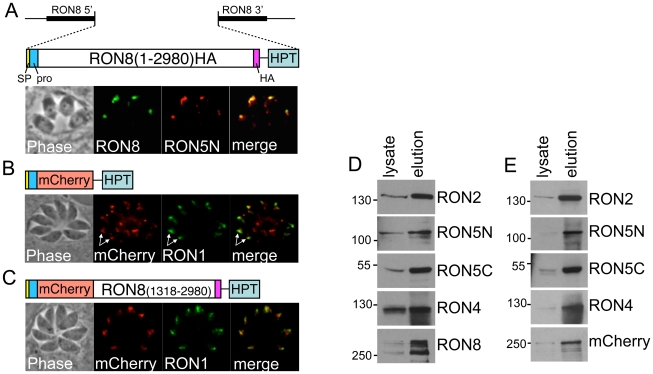
Selective complementation of *Δron8* parasites reveals regions of RON8 necessary for RON8 targeting, complex formation, and function. **A**) Schematic and IFA of complementation with full-length 1–2980 HA-tagged RON8 targeted to the RON8 locus. Shown are the RON8 flanks used for targeting by homologous recombination, and the coding sequence, signal peptide (SP), N-terminal prodomain (pro) and C-terminal HA tag (HA). The selectable marker HPT is also shown. IFA of stable parasite clones shows correct targeting to the rhoptry necks as shown by colocalization with RON5N. **B**) The N-terminal 262 amino acids of RON8 are only partially sufficient for targeting RON8 to the rhoptry necks. The construct contains the first 262 amino acids fused to the mCherry reporter and is targeted to the RON8 locus using the same method as the full length control in “A”. IFA shows that some of the fusion is trafficked properly to the rhoptry necks (arrows) as assessed by colocalization with RON1. However, a significant amount of mistargeted material is also seen in punctate spots in the apical end of the parasite and also in more diffuse patches in the posterior portion of the parasite. **C**) Addition of the C-terminal portion of RON8 (residues 1318–2980) restores rhoptry neck targeting. Diagram of the construct and IFA showing restoration of rhoptry neck targeting upon inclusion of the C-terminal region of the protein. Colocalization is shown using antisera against RON1. **D**) Immunoprecipitation of the MJ complex from parasites expressing the full length RON8 in “A” shows efficient purification of all members of the MJ complex. Western blots show an enrichment of all members compared to whole cell lysates (equivalent amounts of lysate and elution are loaded for each). Anti-RON2 antibodies are used for the immunoprecipitation. **E**) The R8_pro_mCherryR8C fusion is incorporated into the MJ complex. Immunoprecipitation of the MJ complex with anti-RON2 precipitates the R8_pro_mCherryR8C fusion as well as other members of the MJ complex. Thus, the RON8 prodomain plus C-terminal region are sufficient for rhoptry neck targeting and complex association.

Our success in rescuing *Δron8* parasites encouraged us to use selective complementation of the knockout to discern RON8 functional domains as well as regions necessary for trafficking to the neck subcompartment. As RON8 has been shown to have a N-terminal prodomain and such prodomains are known to function in rhoptry targeting, we first tested whether the first 262 amino acids are sufficient for targeting to the rhoptry necks by fusing this region to the reporter protein mCherry (R8_pro_mCherry). We expressed the fusion and a full-length control by targeting each back to the ablated RON8 locus in an expression cassette driven from the RON8 promoter (Figure 5A, B). In stably transformed parasite clones, the R8_pro_mCherry fusion only partially targeted to the rhoptry necks as assessed by colocalization with the rhoptry neck protein RON1 whereas the full-length control targeted perfectly. mCherry that was not localized to the rhoptry necks was found in punctate spots that localized in both apical and basal regions of the parasite. This data suggests that the RON8 prodomain does play some role in trafficking, but additional sequences are also needed for efficient targeting of the protein to the rhoptry necks.

We then assessed whether addition of the C-terminal half of the protein would improve targeting by fusing this portion to the C-terminus of the R8_pro_mCherry protein (Figure 5C). Addition of the C-terminal region of RON8 to the fusion construct (termed R8_pro_mCherryR8C) restored efficient rhoptry neck targeting as seen by colocalization with RON1. Having completely restored rhoptry neck targeting, we determined whether the R8_pro_mCherryR8C fusion was incorporated into the MJ complex by immunoprecipitating the complex with RON2 antisera from parasites expressing the fusion protein, using parasites rescued with the full length protein as a control (Figure 5D/E). Both the control and the R8_pro_mCherryR8C fusion were efficiently co-precipitated with RON2 and the other RONs in the MJ complex. Thus, the RON8 N-terminal prodomain combined with the C-terminal region is sufficient for rhoptry neck targeting and MJ complex formation. While trafficking and complex association were restored, parasites expressing R8_pro_mCherryR8C showed the same defect in invasion as Δ*ron8* parasites (not shown), demonstrating that the N-terminal region of RON8 is necessary for function.

### RON8-deficient parasites are dramatically weakened in virulence *in vivo*

**Figure 6:**
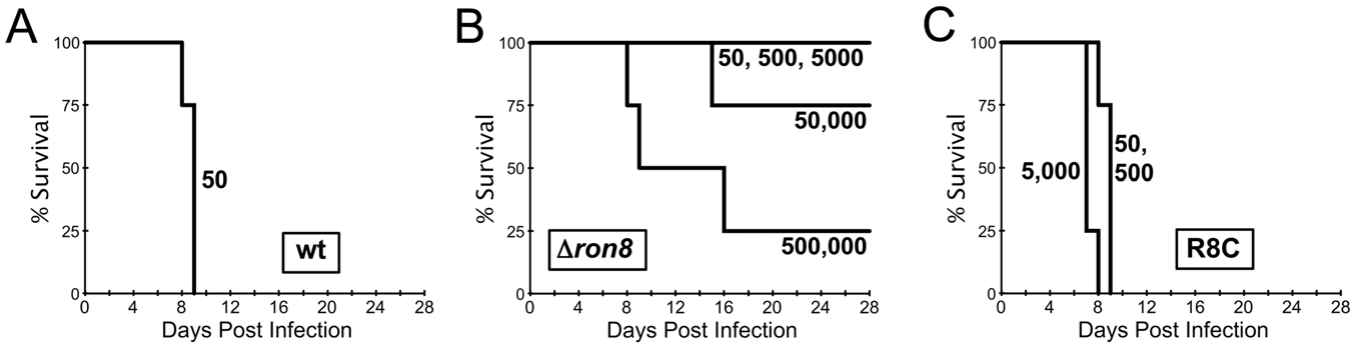
RON8-deficient parasites are severely compromised in establishing disease *in vivo*. **A–C**) Groups of 4 CD1 mice were infected with (**A**) 50 wildtype, (**B**) 50, 500, 5×10^3^, 5×10^4^, or 5×10^5^ *Δron8*, or (**C**) 50, 500, or 5×10^3^ R8c parasites and monitored for 28 days. All surviving mice from *Δron8* parasites were protected against a lethal challenge of 10^4^ wildtype parasites (not shown).

With the significant defects in host cell entry observed *in vitro*, we assessed the degree to which virulence was affected in parasites lacking RON8. To that end, CD1 mice were infected with either a sufficiently lethal dose of wildtype parasites (LD_100_ = 1) or increasing doses of either *Δron8* or R8c (Figure 6). While all mice infected with a low dose of 50 wildtype parasites succumbed to infection by day 9, mice subjected to doses of <5×10^4^ knockout parasites did not show any visible signs of infection. Mice infected with 5×10^4^ parasites did show symptoms of infection, but three of the four mice survived the acute infection. The mice generally became moribund with 5×10^5^ parasites injected; however, even at this high dose, one animal out of the group of four recovered. The virulence phenotype was completely reversed in the complemented strain, demonstrating that the defect was specifically due to the lack of RON8. Mice infected with even the lowest doses of *Δron8* parasites developed an immune response against the parasite as seen by seroconversion, and all mice infected with the knockout survived a challenge with 1×10^4^ wildtype tachyzoites (data not shown). Statistical analysis of these virulence experiments yields an LD_50_ for *Δron8* parasites = ∼2.6×10^5^ parasites, a decrease of more than five logs compared to the wildtype strain. These experiments demonstrate that the inability of *Δron8* parasites to firmly attach and thereby commit to invading host cells *in vitro* produces a dramatically impoverished ability to cause disease *in vivo*.

## Discussion

RON8 is the first moving junction rhoptry neck protein to be functionally analyzed by direct knockout in an apicomplexan parasite. This overturns the previous paradigm that all MJ components are essential for parasite survival, which had been postulated with the need for conditional approaches to study AMA1 [22] and technical difficulties in ablating other junction partners [8], [11]. Deleting RON8 in the *Δku80* strain reinforces both the major technical advance in promoting homologous recombination in parasites lacking KU80 and the potential produced by this advance to examine the moving junction with greater capacity than previously thought. Our success in ablating RON8 is a first step toward ultimately establishing the “minimal” junction complex required by *T. gondii* for host cell entry. AMA1 is already known to be essential for tachyzoite invasion [22]. RON2, which displays a privileged association with AMA1 and likely spans the host membrane [9], will probably also prove to be essential in *Toxoplasma*. This leaves the soluble junction components RON4 and RON5 (which is processed into N and C-terminal fragments), which we are currently attempting to knockout from *Δku80* and *Δron8* parasites to explore their roles in invasion.

RON8's dispensability in *Toxoplasma* agrees with increasing evidence suggesting the moving junction is constructed using different complements of junction proteins deployed during particular life cycle stages in apicomplexan parasites. RON4 can be detected in the ring-shaped junctions of egressing parasites, but AMA1 is absent from these structures, and parasites depleted of AMA1 expression are crippled in invasion, but not affected in egress [8]. Proteomic analysis of *Eimeria tenella* suggests that its ortholog of RON5 is expressed in sporozoites but not in merozoites, while sporozoites appear to lack AMA1 and RON4 [30]. Similarly, PfRON2 is expressed in all *Plasmodium* invasive forms except for ookinetes, which do not form moving junctions and lack rhoptries [31]. Distinct arsenals of junction proteins tailored for specific life cycle stages within each apicomplexan may reflect the evolutionary fine-tuning of this structure that enables phylum members to exploit unique host niches. Our attachment and invasion experiments (Figure 3) suggest that RON8 may have evolved within the coccidia to anchor the invading parasite to common host cytoskeletal proteins [11], although a biochemical demonstration of RON8's link with the host cell will firmly establish this scenario.

**Figure 7:**
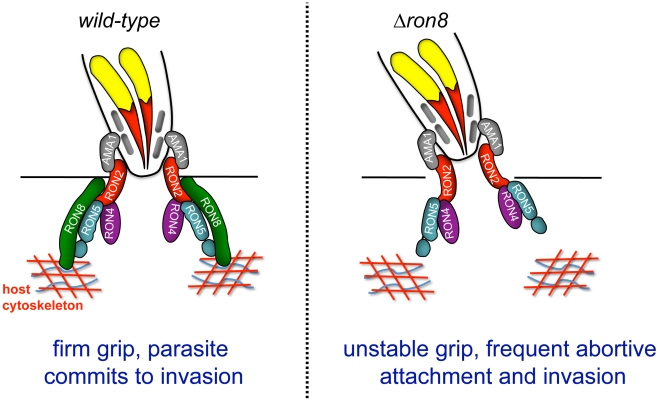
Model of the *Toxoplasma* moving junction in wildtype and Δ*ron8* parasites. The model shows a diagram of a partially invaded parasite with moving junction proteins at the interface of the host and invading parasite. The transmembrane proteins RON2 and AMA1 form the bridge between the host cell and the invading parasite with the soluble proteins RON4, RON5 (processed into N and C fragments) and RON8 exposed to the host cell cytoplasm. RON8 forms a stable intracellular clamp that commits the parasite to invasion, potentially by binding host elements in the cortical cytoskeleton. In the absence of RON8, the MJ is frequently unstable, leading to frequent abortive attachment and invasion.

 The current model of *Toxoplasma* invasion presents attachment as a series of steps increasing in strength, beginning with low affinity interactions between GPI-anchored parasite surface antigens and unknown host contacts, followed by host surface protein association with transmembrane microneme proteins on the parasite surface, and finally moving junction formation through AMA1's interaction with RON2 embedded in the host membrane [9], [19]. The work presented here adds a further critical step to invasion: the secure connection of the parasite to the host through RON8 contacts *within* the host cell, supported by the topology of the RON proteins in the MJ, the exogenous expression of RON8 in mammalian cells [9], [19], and the localization of host F-actin rings observed at *Toxoplasma* and *Plasmodium* moving junctions [32]. According to this updated model (Figure 7), the moving junction in wildtype parasites (Figure 7, *left*) serves as a two-fold lock both inside and outside the host cell, irreversibly directing the parasite toward complete penetration of its target. In knockout parasites (Figure 7, *right*), the loss of the intracellular clasp provided by RON8 severely weakens the moving junction's hold on its target, resulting in frequent detachment from host cells and thereby debilitating the invasive capacity of *Toxoplasma*.

 In addition to disrupting contacts with host components important for parasite adherence, the loss of RON8 imparts further structural abnormalities in the moving junctions of parasites that overcome this defect, as evidenced by the appearance of MJ components in trails dragging behind newly invaded *Δron8* parasites (Figure 4B, C). Disorganized trails are not ubiquitous during pulse invasion of these parasites (∼20%), suggesting that the structure is not entirely disorganized, a finding which is supported by the recovery of the remainder of the junction complex by co-immunoprecipitation (Figure 5). Junctions that are compromised enough to form trails could indicate difficulties in PVM separation from the host membrane without RON8, and the low numbers of evacuoles observed in cytD-arrested *Δron8* parasites suggests RON8 function is a prerequisite for rhoptry bulb secretion. The latter phenotype is reminiscent of defects observed in parasites depleted of AMA1, which reorient after initial contact with the host cell but generally do not form a moving junction [8] or inject evacuoles [22]. Similarly, preventing AMA1-RON complex formation using inhibitory peptides abolishes evacuole secretion in *Plasmodium* [19]. While this indicates some signal occurs after microneme and rhoptry neck secretion to allow rhoptry bulb secretion, it is unclear how such a signal is transmitted between apical organelles of the invading parasite.

 We have demonstrated that RON8 is processed at its C-terminus (Figure 2D) at a site that cannot be readily resolved by Western analysis comparing the size of the HA-tagged RON8 precursor with its mature form. This cleavage event is distinct from RON8's processing at the N-terminus demonstrated in Besteiro et al [9], which also showed all MJ proteins are subject to proteolysis, probably at ROP1-like SφXE sites which are believed to be processed by the subtilisin protease TgSUB2 [33]. There are two candidate processing sites near the C-terminus that match this consensus site (SAME at residues 2652–2655 and SAGE at 2702–2705), but cleavage at these sites would remove ∼30–35 kDa which might be resolvable by SDS-PAGE. Additional sites are also possible as we have seen some minor variations in the residues present in these cleavage sites (Hajagos and Bradley, unpublished results) and others have highlighted a potential cleavage site at the extreme C-terminus of RON8 that would remove only nineteen amino acids from the protein (residues 2958–2961, SFLQ, [9]). Our HA-tagged construct and additional fusions will undoubtedly aid in determining the precise cleavage site and the role of processing in RON8 targeting, complex formation and function.

RON8's exposure to the host cytosol during invasion makes it a prime candidate for restricting access of host transmembrane proteins to the nascent PVM, which may occur in conjunction with the stabilizing grip on host components identified in this work. Our initial attempts to examine the ability of *Δron8* parasites to exclude host Na^+^/K^+^ ATPase (known to be sieved during invasion) by immunofluorescence has not revealed any gross differences from the wildtype strain, although minor changes would be difficult to detect (data not shown). While we cannot exclude a role for this protein in some aspect of molecular sieving, it is possible that RON8's sole function is to firmly grip host peripheral components to completely anchor the parasite, leaving other cytosolic-exposed junction proteins (RON4 and RON5) to conduct filtration.

 It is currently unknown whether the individual RON proteins of the MJ complex each contain their own rhoptry neck targeting information or if one protein can escort the others as is seen in adhesive complexes secreted from the micronemes and for the *Plasmodium* rhoptry protein RAP1 [34], [35], [36]. While the precise cleavage site of RON8's N-terminal prodomain has not been determined, this region does appear to contain some but not complete rhoptry targeting information. Addition of the C-terminal region fully restores rhoptry neck targeting, which is likely due to the ability to associate with other complex members (Figure 5E). The lack of a functional rescue in parasites lacking the N-terminal region of RON8 indicates that this region contains domains that are critical for invasion. These domains could play a direct role in the mechanics of invasion or provide additional interaction domains, thus strengthening the integration of RON8 into the complex. Deletion of the N-terminal region could also impact folding of the remaining C-terminal portion in the complex, which our previous exogenous expression data suggests is binding to host components at the periphery of the cell [11]. While a large number of functional domains are likely to be present in the 330 kDa RON8 protein, the data presented here demonstrate that functional complementation promises to be a powerful tool to further dissect specific regions of RON8 involved in targeting, complex formation, and function.

We have shown that the RON8-deficient strain is more than five logs less virulent than wildtype *Δku80* parasites in outbred CD1 mice (Figure 6). The contribution of MJ/RONs to the host response during *in vivo Toxoplasma* infection is likely minimal, due to the short period of time the moving junction is present during invasion (∼30 sec) as well as the topology of RON8 and other soluble MJ/RONs beneath the plasma membrane in this structure [9], [11]. Studies of RON4 antigenicity in *Plasmodium falciparum* [37] and *P. yoelii* [18] support this, as this protein displays extensive sequence conservation with concordantly little apparent immune pressure. The dramatic impact on virulence we observe in *Δron8* parasites is therefore in all likelihood a consequence of the significant defects in host cell entry displayed *in vitro* rather than any enhanced host reaction to the parasite.

In conclusion, we have utilized RON8 knockouts to make greater inroads into analyzing rhoptry neck protein function than previously achieved in *Toxoplasma*. In the process, we have expanded the model of *T. gondii* invasion by implicating at least one rhoptry protein in ensuring the parasite's commitment to entering its target cell. Our complementation strategy will enable further dissection of RON8 functional and interaction domains, which promises a more detailed understanding of the molecular architecture of this phenomenal structure. Future experiments will also examine RON8's contribution to egress, as moving junctions are observed during pathogen exit from the host cell [8]. Although RON8 is a coccidia-specific member of the complex, identifying host contacts with this protein will likely prove useful in preventing invasion across the phylum, as host proteins regulating cytoskeletal filament assembly are recruited to the junctions of invading *Plasmodium* and *Toxoplasma* [32]. Through this work and upcoming studies, a complete understanding of the moving junction's participation in *Toxoplasma*'s remarkable success at a parasitic lifestyle will be at hand.

## Materials and Methods

### Ethics statement

*Toxoplasma* infections in mice and antibodies raised in mice were performed under the guidelines of the Animal Welfare Act and the PHS Policy on Humane Care and Use of Laboratory Animals. Specific details of our protocol were approved by the UCLA Animal Research Committee (ARC# 2004-055).

### Parasite and host cell culture

The RHΔ*hpt* and RHΔ*hptΔku80* strains of *Toxoplasma* have been previously described [25], [38], and were maintained on confluent monolayers of human foreskin fibroblasts (HFFs) grown in Dulbecco's modified eagle medium supplemented with 5% fetal bovine serum, 5% Cosmic Calf Serum (Hyclone), and 2 mM glutamine [12].

### Antibodies and Western blot analysis

The following antibodies were used in immunofluorescence and Western blot assays: mouse polyclonal anti-RON8 (1∶400) [11], rabbit polyclonal anti-RON4 (1∶7000) [8], mouse polyclonal anti-RON2 (1∶800) [12], rabbit polyclonal anti-RON2 (1∶1000) (described below), mouse polyclonal anti-RON5N and RON5C (both 1∶600) [11], mouse polyclonal anti-RON1 (1∶300) [8], mouse monoclonal anti-ISP1 [25], rabbit polyclonal anti-SAG1 (1∶100000), a gift from John Boothroyd, rabbit polyclonal anti-SAG2 (1∶4000, John Boothroyd), mouse monoclonal T34A7 against ROP2/3/4 (1∶300) [39], rabbit polyclonal anti-ROP13 (1∶1000) [40], mouse monoclonal 1B10 anti-ROP7 (1∶1000) [41], rabbit polyclonal anti-pro-ROP4 (1∶1000) [26], rabbit polyclonal anti-HA (1∶300) (Invitrogen), and mouse monoclonal anti-HA (1∶500) (Covance). SDS-PAGE gels were used to resolve proteins by Western blot analysis as previously described [42]. Secondary antibodies were horseradish peroxidase (HRP)-conjugated goat anti-mouse and goat anti-rabbit used at a dilution of 1∶500–2000 (Sigma) and detected using the ECL Western Blot Detection Kit (Thermo Scientific).

To generate a RON2 fusion protein with a N-terminal 6xHis tag for antibody production, the *RON2* cDNA encoding amino acids 25 to 313 (residue numbers are from Genbank accession HQ110093 with residue 1 as the start methionine) was amplified from a *Toxoplasma* RH strain cDNA library using primers P21 and P22 (Table S1). The amplified product was cloned into *pET28a(+)* (Novagen) using the HindIII and XhoI sites encoded in the primers. Production and purification of rRON2_25–313_ from *E. coli* strain Rosetta (Novagen) using a nickel-nitrilotriacetic acid matrix were done essentially following manufacturer's instructions (Qiagen). Antibodies to rRON2_25–313_ were generated in rabbits by Covance, Inc.

Specific antisera against mCherry (used at 1∶4000) was generated in mice using recombinant 6xHis tagged protein purified by denaturing nickel agarose chromatography. mCherry was amplified using primers p23 and p24 and the amplified product was cloned into the pET101 (Invitrogen) bacterial expression plasmid which encodes a 6xHis tag in frame with the C-terminus of the gene. BL21-DE3 cells were transformed with the construct and induced with 1 mM IPTG for 5 hours before the cells were collected and mCherry-6xHis was purified and dialyzed against PBS. BALB/c mice (Charles River) were immunized with ∼70 µg of recombinant protein on a 21 day immunization schedule. The resulting mouse polyclonal antiserum were collected and tested by Western blot analysis.

### Disruption of RON8 in *T. gondii*

The initial *RON8* KO vector was previously described [11]. The construct was linearized by KpnI digestion, and 30 µg of DNA were transfected by electroporation into Δ*ku80xΔhpt* strain parasites [25]. Following selection of transformants in media containing 50 µg/ml MPA and 50 µg/ml xanthine for five days, the knockout populations were scrape-syringed and cloned by limiting dilution. GFP negative parasites were screened by immunofluorescence using anti-RON8 polyclonal antisera, and a clonal line of GFP/RON8 double negative parasites was named strain *Δron8* (*1-1716, +HPT*). For generation of the *HPT KO* vector, the HPT cassette from *RON8 KO* was removed by digestion with XbaI and re-ligated, generating *RON8KOΔHPT*. To remove the remaining RON8 coding region along with the HPT marker, a new 3′ flank downstream of the *RON8* stop codon was amplified from RH strain genomic DNA using primers P9 and P10 and subcloned into *RON8KOΔHPT* using KpnI and XbaI to make *HPT KO*. This plasmid was linearized by KpnI digestion and 50 µg of DNA transfected by electroporation into *Δron8* (*1-1716, +HPT*) parasites. Exclusion of *HPT* was selected for using 350 µg/ml 6-thioxanthine (Sigma) for 5 weeks, after which populations were cloned by limiting dilution. Clones were then screened for the absence of RON8 and susceptibility to medium containing 50 µg/ml MPA and 50 µg/ml xanthine, to identify *Δron8* parasites.

### Generation of full-length and selectively complemented *Δron8* parasites

An *HPT* cassette was amplified from the pMini-GFP.ht plasmid [43] using primers P11 and P12 and cloned into pCR2.1-TOPO (Invitrogen) as per manufacturer's instructions. Sequences upstream (using primers P13 and P14) and downstream (primers P15 and P16) of the ablated *KU80* locus were amplified from RH*ΔhptΔku80* genomic DNA and subcloned into pCR2.1-TOPO+*HPT* using KpnI and SpeI for the 5′ flank (∼1.5 kb) and NsiI and XbaI for the 3′ flank (∼1.1 kb). The 12 kb RON8-HA expression cassette was subcloned into this vector at an EcoRV site following excision from *pGRA-HA_HPT-RON8* [11] by PciI and DraIII digestion and blunting with Klenow fragment (NEB). Sequencing established the inverse orientation of the cassette relative to the *KU80* flanks and confirmed placement of the *HPT* and RON8-HA cassettes between these flanks. This vector (*R8compHPT*) was then linearized by PmeI digestion, and 25* µ*g of DNA transfected into *Δron8* parasites, followed by MPA/xanthine selection. Resistant clones were screened by IFA and RON8 complemented parasites were confirmed by Western blot analysis, identifying R8c strain parasites.

The *HPT* cassette described above was also subcloned back into the *HPT KO* vector after blunting at the XbaI site. This vector, *HPTKO+HPT*, was then digested by NotI and ApaI, blunted, and ligated with the blunted 12 kb RON8 expression cassette described above to make a complementation vector encoding *HPT* and RON8-HA adjacent to RON8 flanks lying outside the entire RON8 coding sequence. After confirming forward orientation of the RON8-HA cassette relative to the *RON8* flanks and placement of the *HPT* and RON8-HA cassettes between these flanks by sequencing, this vector (*R8HPTKO+HPT*) was linearized by AflII digestion and transfected (25 µg) into *Δron8* parasites, followed by MPA/xanthine selection for 9 days and cloning. Resistant clones were screened by IFA and RON8 complemented parasites were confirmed by Western blot analysis, with positive clones named *Δron8 + RON8_1–2980_* parasites.

To make fusions of RON8 fragments with mCherry, primers P17 and P18 were used to amplify the mCherry coding sequence using pmCherry Vector (Clontech) as a template. This PCR product contains SmaI at one end and AvrII/PacI sites on the other end; after SmaI/PacI digestion, it was subcloned into *R8HPTKO+HPT* digested at SmaI and PacI endogenous sites in RON8-HA. This replaced sequences encoding residues 263–2980 and the HA tag with mCherry. This vector (*R8proMCHERHPTKO*) was linearized with AflII and 25 µg of DNA transfected into *Δron8* parasites prior to 9 days MPA/xanthine selection, cloning, and IFA screening as above, generating R8_pro_mCherry parasites.

For introduction of the RON8 C-terminus into *R8proMCHERHPTKO*, the sequence encoding residues 1318–2980 and the C-terminal HA tag flanked by AvrII/PacI sites was amplified from template pGRA-HA_HPT-RON8 using primers P19 and P20. This fragment was subcloned into *R8proMCHERHPTKO* at AvrII/PacI sites, sequenced to confirm both junctions with mCherry were in frame, linearized with AflII, transfected, drug selected, cloned, and screened by IFA as above to identify *Δron8 +* R8_pro_mCherryR8C parasites.

### PCR amplification for verification of constructs and knockouts

Template DNA was extracted from harvested wildtype *Δku80*, *Δron8*, or R8c parasites using the Wizard Genomic DNA Purification Kit (Promega) as per the manufacturer's protocol. RON8 coding sequence removal in *Δron8* parasites was confirmed using primers P1 and P2 using either *Δku80* or *Δron8* genomic DNA. The removal of the entire *RON8* genomic locus with concomitant bridging of flanking sequences normally separated by ∼20 kb was confirmed using primers P3 and P4 using *Δku80*, *Δron8*, or R8c genomic DNA as a template. Correct targeting of the full-length RON8 complementation cassette at the *KU80* locus was confirmed using primers P5 with P6 and P7 with P8.

### Immunofluorescence and invasion assays

Intracellular parasites were examined by immunofluorescence as per [11]; confluent HFFs grown on glass coverslips were infected with parasites and incubated for 24–30 hours at 37°C, washed with PBS, and then fixed with either ice-cold methanol for 3 minutes or 3.7% formaldehyde/PBS for 15 minutes prior to quenching with phosphate-buffered saline (PBS) plus 0.1 M glycine for 5 min. Coverslips were then washed in PBS and blocked with PBS/3% bovine serum albumin (BSA) or a completely permeabilizing solution of PBS/3%BSA/0.1% Triton X-100 (PBT buffer) for 30 minutes. Primary antibodies were diluted in PBS/3%BSA or PBT buffer for 1 hour. Coverslips were washed five times in PBS and incubated with secondary antibodies Alexa-488 goat anti-mouse and Alexa-594 goat anti-rabbit (or Alexa 594 goat anti-mouse and Alexa 488 goat anti-rabbit, Molecular Probes, OR) diluted 1∶2000 in PBS/3%BSA for 1 hour. Following secondary washes in PBS, coverslips were then mounted onto slides using Vectashield mounting medium for fluorescence microscopy using a Zeiss upright light microscope (Zeiss Axio Imager Z1) using either a 100x or 63x oil immersion objective. All images were rendered using Axiovision software in conjunction with a Zeiss digital CCD camera (AxioCam MRm). Early invasion experiments were performed using low temperatures as per [11].

Invasion assays were conducted to distinguish between extracellular parasites and internalized parasites via a red/green invasion assay as described [27]. In brief, equivalent cultures of wildtype *Δku80*, *Δron8*, and R8c parasites were scraped and passed through a 27-gauge needle to collect strictly intracellular parasites for use in the invasion assay. After washing with fresh medium, ∼3×10^6^ tachyzoites were resuspended in 500 µl of fresh prewarmed DMEM and placed onto separate chambers of an 8-well chamber slide (Falcon) containing confluent HFF monolayers. These parasites were allowed to invade for one hour at 37°C, after which monolayers were washed three times in PBS and fixed with EM-grade 3.7% formaldehyde/PBS (Biosciences, Inc.). After quenching as above, the samples were blocked in PBS/3%BSA for 25 minutes and incubated with rabbit anti-SAG1 diluted in PBS/3%BSA for 1 hour, then washed five times in 1X PBS and permeabilized with PBT buffer for 30 minutes prior to incubation with mouse anti-ROP7 diluted in PBT buffer as a second primary step. Secondary staining and fluorescence microscopy then proceeded as above. Parasites staining with both anti-SAG1 and ROP7 denoted attached but uninvaded parasites, while those staining only for ROP7 were scored as internalized. Nine fields were randomly counted for each chamber, yielding total counts of 250–800 parasites for all strains. Invasion experiments were conducted in triplicate and repeated at least twice. To examine whether the Δ*ron8* parasites could be rescued by host cells exogenously expressing RON8, we used a competition growth assay in cells with and without RON8 expression. No difference in the rate at which the wildtype parasites outcompeted the knockout was observed, indicating that exogenously expressed RON8 cannot complement the invasion defect.

Attachment and evacuole assays utilizing cytochalasin D were performed as described [22]; wildtype, *Δron8*, and R8c parasites were scrape-syringed as above and resuspended in Endo buffer (44.7 mM K_2_SO_4_, 10 mM Mg_2_SO_4_, 106 mM sucrose, 5 mM glucose, 20 mM Tris, 0.35% wt/vol BSA, pH 8.2) containing 1 µM cytochalasin D (Sigma-Aldrich). After incubation at room temperature for 10 min, ∼3×10^6^ parasites from each strain were used to infect HFF monolayers grown on 8-chamber slides and incubated for 20 min at 37°C. Media was replaced with prewarmed DMEM/10% FBS containing 1 µM cytochalasin D and incubation continued for another 15 min at 37°C before fixation with formaldehyde and immunofluorescence/counting as described above for red/green invasion assays (except that instead of anti-ROP7 antibody, evacuoles were labeled with monoclonal anti-ROP2/3/4 antibody).

To examine whether MJ component trails are present in extracellular parasites, intracellular wildtype or *Δron8* parasites were scrape-syringed, washed once in PBS, and allowed to adhere to slides prior to coating with 3.7% formaldehyde/PBS fixative for 15 min prior to quenching with 0.1 M glycine/PBS as above. Following fixation, blocking/permeabilization in PBT buffer and staining with rabbit anti-RON4 was performed as before.

### Coimmunoprecipitation of the MJ complex from complemented *Δron8* strains

Rabbit polyclonal anti-RON2 was cross-linked to Protein A-Sepharose beads (Amersham) using dimethyl pimelimidate as described [8]. RON2-linked beads were then incubated with parasite lysates made in a modified RIPA buffer + Complete Protease Inhibitor described in [8] (50 mM Tris-Cl pH 8.0, 5 mM EDTA, 75 mM NaCl, 1% NP40, 0.5% DOC, 0.005% SDS). In brief, ∼2×10^8^ extracellular parasites were centrifuged at 3000 *g* for 20 min. The parasites were washed once in 1X PBS and then lysed on ice for 20 min prior to removing insoluble material by centrifugation at 10000 *g* for 20 min. Antibody-coupled beads were incubated with lysate at 25°C for 3 hours before four washes with lysis buffer. The bound proteins were eluted using 100 mM triethylamine pH 11.5, and lyophilized to remove the triethylamine and concentrate the eluate. The resulting products were analyzed by Western blot using antibodies against RON/MJ proteins.

### Gliding motility trail assays

Motility experiments were performed largely as described [44]; wildtype or *Δron8* parasites were resuspended in Hank's buffered saline solution (HBSS) and allowed to glide on serum-coated glass coverslips for 20 min at 37°C. Coverslips were rinsed twice in PBS and fixed with EM-grade formaldehyde for 15 min prior to immunofluorescence with rabbit anti-SAG2 antibodies as described above.

### Mouse virulence assays

Intracellular wildtype (Δ*ku80*Δ*hpt* parental), Δ*ron8*, and R8c parasites were scrape-syringed from infected HFF monolayers and resuspended in Opti-MEM prior to intraperitoneal injection of 50 wildtype (Δ*ku80*Δ*hpt* parental); 50, 500, 5×10^3^, 5×10^4^, or 5×10^5^ *Δron8*; or 50, 500, or 5×10^3^ R8c strain tachyzoites in outbred CD1 female mice, making a total of 9 groups of 4 mice each. Mice in each group were bled both prior to injection and surviving mice bled 15 days post infection, and serum used in Western blot analysis lysates from wildtype parasites to test for seroconversion. Prism GraphPad software was used to determine the LD_50_ of *Δron8* parasites from an analysis of the results by a standard sigmoidal dose-response curve. Mice were monitored for 25 days, and surviving mice “protected” by *Δron8* immunization were challenged by intraperitoneal injection with 1×10^4^ wildtype tachyzoites at day 30 and assessed for an additional 30 days. All care and handling of animals was in accordance with institutional guidelines and approved by the UCLA Animal Research Committee.

## Supporting Information

Figure S1 Gross assessment of microneme function by examining MIC2 protein levels and gliding motility in Δ*ron8* and control parasites. **A**) IFA of wildtype and Δ*ron8* parasites showing no noticeable change in localization of MIC2. **B**) Western blot analysis of total parasite lysates from wild-type, *Δron8*, and R8c parasites showing approximately even levels of MIC2 protein are present. RON2 is used as a loading control. **C**) Ethanol induced secretion shows no apparent change in secreted MIC2. Parasite equivalents are shown for each sample and are used as a loading control showing that ∼33% of the MIC2 is released from both wildtype and *Δron8* parasites. **D**) Gliding motility assays staining for SAG2 deposited on serum-coated glass coverslips. Similar trails were observed for wildtype and knockout parasites showing that motility is not substantially compromised in *Δron8* parasites.
(TIF)

Table S1 Oligonucleotide primers utilized in this study.
(DOC)
