# Supplementary figures and images for: The Moving Junction Protein RON8 Facilitates Firm Attachment and Host Cell Invasion in Toxoplasma gondii
Source: PLoS Pathog. 2011 Mar 10;7(3):e1002007. doi: 10.1371/journal.ppat.1002007 (PMC3053350; doi:10.1371/journal.ppat.1002007)

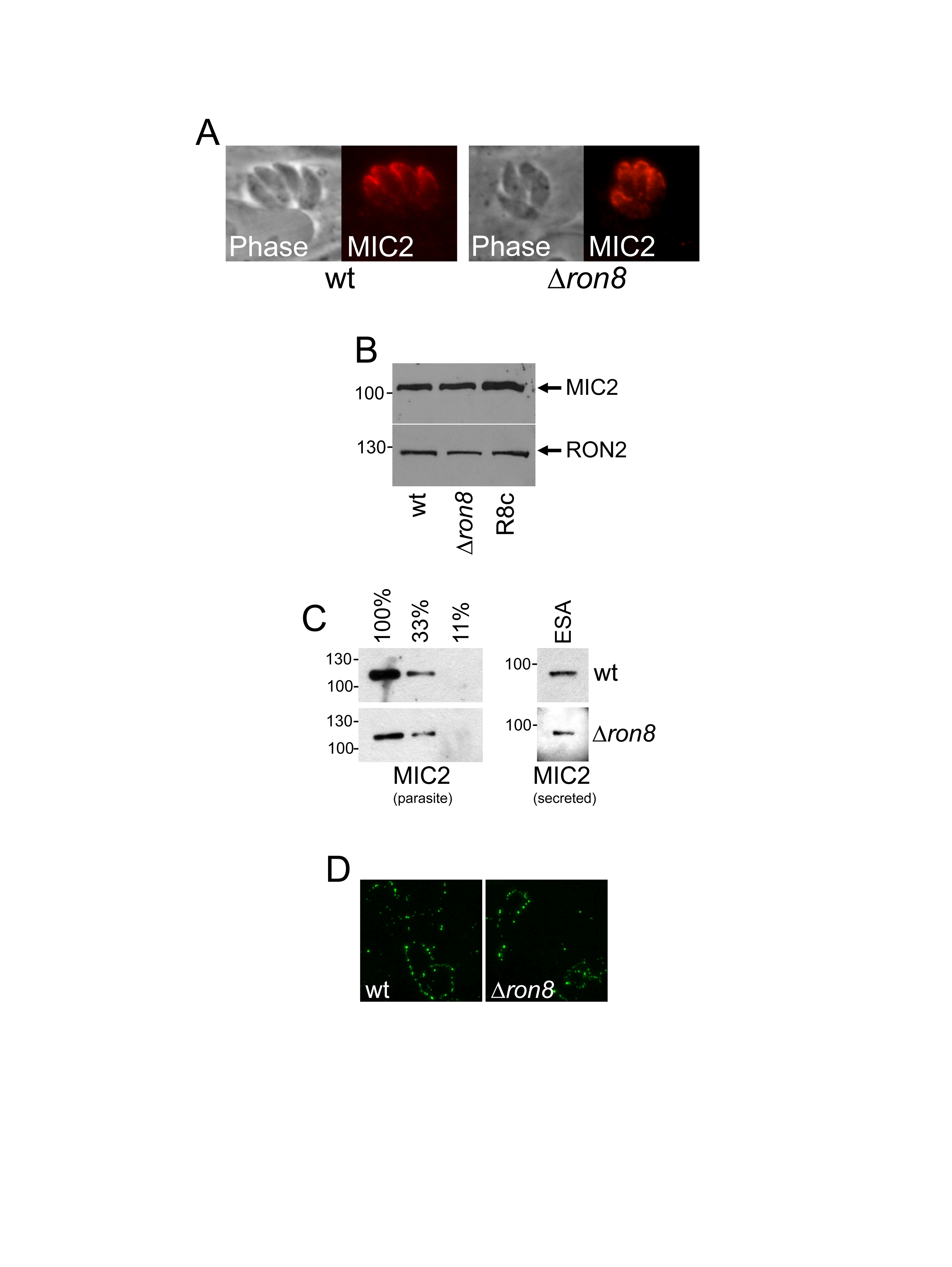

Supplement: Figure S1 — Gross assessment of microneme function by examining MIC2 protein levels and gliding motility in Δron8 and control parasites. A) IFA of wildtype and Δron8 parasites showing no noticeable change in localization of MIC2. B) Western blot analysis of total parasite lysates from wild-type, Δron8, and R8c parasites showing approximately even levels of MIC2 protein are present. RON2 is used as a loading control. C) Ethanol induced secretion shows no apparent change in secreted MIC2. Parasite equivalents are shown for each sample and are used as a loading control showing that ∼33% of the MIC2 is released from both wildtype and Δron8 parasites. D) Gliding motility assays staining for SAG2 deposited on serum-coated glass coverslips. Similar trails were observed for wildtype and knockout parasites showing that motility is not substantially compromised in Δron8 parasites. (TIF) [file ppat.1002007.s001.tif]
