# Supplementary material for: The Moving Junction Protein RON8 Facilitates Firm Attachment and Host Cell Invasion in Toxoplasma gondii
Source: PLoS Pathog. 2011 Mar 10;7(3):e1002007. doi: 10.1371/journal.ppat.1002007 (PMC3053350; doi:10.1371/journal.ppat.1002007)
Supplement: Table S1 — Oligonucleotide primers utilized in this study. (DOC) [file ppat.1002007.s002.doc]

# Table 1. Oligonucleotide primers utilized in this study.

| Name | Primer Sequence |
| --- | --- |
| P1 | CACCGTTAACCACCACCATGGGCCATCCGTCATTGGGGCACTGG |
| P2 | CACCGCGGCCGCGATACCTCTATGCTGCCGAAG |
| P3 | GCCAGGTACCGCTGTAGGCGGAGAATTGTTC |
| P4 | AGTAGCGGCCGCCAGCAAGATGTCTCGATGTAC |
| P5 | GGAAGTAACATGCCTCTTGTTC |
| P6 | GGTCTCGATGTCGAACAAAGC |
| P7 | GCGCTCTAGAGCGATGCAAGACGATTCAGGC |
| P8 | CACTCATGAAAACGGTGCTGC |
| P9 | GGTACCGCTGTAGGCGGAGAATTGTTC |
| P10 | TCTAGAGTGTCTCTGTGATGCGATAAG |
| P11 | CATGGAGCTCGAGGTTTACACTTAGGTGGTGCGG |
| P12 | CATCGAGCTCGAGATTCAAACCCGCCCGCGGAAG |
| P13 | CAATGGTACCTTGTTTAAACGCTTAGGCAGACGCAGCGAAC |
| P14 | GTTCACTAGTGAACTTGCTTTGCCCTTGTCG |
| P15 | CTCTATGCATGCAGTTTTCGATTTTCCGTCTC |
| P16 | GAAATCTAGAGGTGCACACAAGCAATGAAAG |
| P17 | GAATCCCGGGGTGAGCAAGGGCGAGGAGG |
| P18 | CCTTTTAATTAACCCCTAGGCTTGTACAGCTCGTCCATGCC |
| P19 | CTATCCTAGGTATGCATTCGTGACACATCTCC |
| P20 | CCTGTTAATTAATCATGCGTAGTCGGGG |
| P21 | cccaagcttgcctcttcttctctagctttcc |
| P22 | ccgctcgagttaTCTCTCCTTTTCTGTCTGGT |
| P23 | CACCATGGTGAGCAAGGGCGAGGA |
| P24 | CTTGTACAGCTCGTCCATGC |
